# Does the addition of metformin to carboplatin treatment decreases ovarian reserve damage associated with carboplatin usage?

**DOI:** 10.1186/s13048-023-01259-2

**Published:** 2023-09-02

**Authors:** Sevgi Ayhan, Necati Hancerliogullari, Gurhan Guney, Murat Gozukucuk, Muzaffer Caydere, Sergul Selvi Guney, Aytekin Tokmak, Yusuf Ustun

**Affiliations:** 1 Department of Obstetrics and Gynecology, University of Health Sciences, Bilkent City Hospital, Ankara, Turkey; 2 Department of Reproductive Endocrinology and Infertility, Balikesir University School of Medicine, Cagis Campus,10145, 10145 Balikesir, Turkey; 3 Department of Obstetrics and Gynecology, University of Health Sciences, Ankara Training and Research Hospital, Ankara, Turkey; 4 Department of Pathology, University of Health Sciences, Ankara Training and Research Hospital, Ankara, Turkey; 5 Department of Midwifery, Faculty of Health Sciences, Balikesir University, Balikesir, Turkey

**Keywords:** Antioxidant, Catalase, Malondialdehyde, Metformin, Carboplatin, Ovarian cancer, AMH

## Abstract

**Background:**

We aimed to determine whether adding metformin to carboplatin treatment would reduce the damage to ovarian reserve associated with carboplatin use.

**Methods:**

We included 35 adult female non-pregnant albino Wistar rats approximately three months old, weighing 220–310 g. The rats were divided into five groups of seven rats according to the treatment they received. Carboplatin and salin was given to Group 2, and carboplatin plus metformin was given to Group 3. Group 4 was administered only metformin. Group 5 was administered only salin. Carboplatin was given to Groups 2 and 3 as a single dose on the 15th day, while metformin was given to Groups 3 and 4 during the 28-day experiment. After oophorectomy, histopathologic analyses of primordial, primary, secondary, and tertiary Graff follicles according to the epithelial cells surrounding the oocyte and total follicular number were conducted per section. Serum Anti-Mullerian Hormone (AMH), tissue catalase, and malonyl dialdehyde levels were measured and compared within each group.

**Results:**

The baseline and 15th-day serum AMH values of the menstrual cycle were compared among the groups, and no statistically significant differences were observed (p > 0.05). Group 3, which was given both carboplatin and metformin, had statistically significantly higher 28th-day AMH levels than Group 2, which was given only carboplatin and saline (p < 0.001). The number of primordial follicles in Group 3 was found to be statistically significantly higher than in Group 2 (p < 0.001). Tissue catalase enzyme levels in Group 3 were statistically significantly higher than in Group 2 (p < 0.001). Tissue malondialdehyde levels in Group 2 were statistically significantly higher than tissue malondialdehyde levels in Groups 3 and 4 (p < 0.001).

**Conclusions:**

Metformin may attenuate carboplatin-induced ovarian damage, possibly through its antioxidative effects.

## Background

Ovarian cancer is a fatal gynecological cancer. In addition, among all cancers, it is the fifth most common cause of death [1]. Since most cases are diagnosed late, the prognosis is poor. Moreover, existing screening tests have low predictive value in diagnosis. The first treatment option for ovarian cancer is complete reduction surgery and platinum-based chemotherapy [2, 3]. Carboplatin (1,1-cyclobutyldicarboxylate) is a second-generation, cell-cycle platinum alkylating agent that contains a platinum atom complex with two ammonia groups and a cyclobutane-dicarboxyl residue. In addition to other solid malignancies, it is frequently used to treat a variety of gynecological cancers, such as ovarian, endometrial, and cervical cancers [4]. The molecular mechanism underlying the antitumor effect of carboplatin depends on its binding to DNA in the nucleus. As a result, it causes cell apoptosis or necrosis by blocking DNA synthesis. Another mechanism of the effect of carboplatin is that it accelerates the apoptosis of cancer cells by increasing the formation of reactive oxygen radicals and creating cytotoxicity through oxidative stress [5]. Because carboplatin affects ovarian tissue, it also affects normal dividing noncancerous cells and causes gonadotoxicity in long-term use. The causes of carboplatin gonadotoxicity are apoptosis, oxidative stress, and DNA repair deficiency, which may lead to the depletion of the primordial follicle, causing damage to ovarian tissue [6]. Although ovarian tissue and oocyte cryopreservation can be successfully applied in pediatric and other patients, these methods are invasive, expensive and not applicable in all situations. Hence, research in this area has continued, and new treatment protocols have recently been developed to prevent ovarian failure, infertility, and other side effects due to adjuvant chemotherapy.

Metformin is a biguanide derivative of oral antihyperglycemic and insulin-sensitizing drugs commonly used in the treatment of diabetes. Metformin improves the prognosis of diabetics not only by lowering blood sugar but also by lowering oxidative stress and cytokine production. The antioxidative features of metformin occur when the balance between antioxidant enzymes and ROS production changes to increasing antioxidant enzyme levels. This phenomenon is mediated by the influence on AMPK activity [7]. AMPK is an energy sensor that is activated by a rise in the ratio of AMP to ATP, or an increase in the amount of AMP in the cell. Metformin activates the AMPK pathway, which results in the inhibition of some pathways that are effective in cancer formation, such as p53 and mTor. These interactions indicate that metformin has anticancer effects as well as antioxidant and anti-inflammatory features. Therefore, metformin is an ideal agent for both cancer treatment and reducing the side effects of cancer treatment [8]. Recently, there has been a significant decrease in the cancer-related deaths of young patients. The issue of fertility preservation has become important in the management of these patients. Beyond the above-mentioned mechanisms, metformin plays an important role in follicle development and ovulation by enhancing insulin sensitivity, regulating various intraovarian growth factors and reducing androgens. Although there are several studies in the literature on the positive effects of metformin on ovarian reserve [9], the number of studies on the protective effects of chemotherapy-induced ovarian damage is limited. In addition, the mechanisms by which metformin improves ovarian function have not yet been clearly shown.

In this study, based on the above relationships, we aimed to investigate whether we could reduce ovarian reserve damage due to carboplatin usage by adding metformin to carboplatin therapy. To evaluate ovarian reserve, we investigated the levels of the anti-Mullerian hormone (AMH), a glycoprotein secreted from granulosa cells in preantral and small antral follicles. AMH is a reliable marker of ovarian reserve assessment, which is not affected by the menstrual cycle [10]. To measure the oxidative damage caused by carboplatin, we also investigated the tissue levels of malonyl dialdehyde, a lipid peroxidation product, and the tissue levels of catalase, which is one of the most important antioxidant enzymes secreted to balance malondialdehyde [11].

## Methods

### Animals and experimental groups

The experiments were performed in accordance with the guidelines in Turkish Central Ethics Committee for Animal Experiments (CECAE). The study was approved by the Ankara Hospital Animal Research Ethics Committee (protocol number: 545/2018). We included 35 adult non-pregnant female albino Wistar rats approximately three months old, weighing 220–310 g. The rats were fed unrestrictedly with standard ram chow in pellet form, in accordance with the national guidelines. All rats were studied in a standard laboratory environment in a 12-h light–dark period at 21–23 °C and 50% relative humidity.

### Drug administration

The rats were divided into five groups of seven rats according to the treatment they received. Group 1 was a sham group that did not receive medical treatment. Group 2 received carboplatin (80 mg/kg) intraperitoneal plus saline, as administered in a previous study [12]. Group 3 received concomitant carboplatin and metformin (50 mg/kg/day), following previous studies performed on rat ovaries [13] (Table 1). Group 4 received only metformin (50 mg/kg/day). Group 5 received saline. Metformin was administered to Groups 3 and 4 for 28 days using the gavage technique. Carboplatin was administered to Groups 2 and 3 in single doses on the 15th day. Saline was administered at 1 mg/kg/day to Groups 2 and 5 for 28 days using the gavage technique.

**Table 1:** Comparison of changes in AMH values over time between groups

	Group 1 (Sh)	Group 2 (CP + Saline)	Group 3 (CP + M)	Group 4 (M)	Group 5 (S)	p^*^
AMH basal	8.5 ± 0.7	8.3 ± 0.5^e^	8.3 ± 0.9	8.5 ± 0.7	8.4 ± 0.5	> 0.05
AMH 15th day	8.7 ± 1.0	8.3 ± 0.8^e^	8.6 ± 1.0	8.8 ± 1.0	8.6 ± 0.9	> 0.05
AMH 28th day	8.7 ± 0.8^a^	5.3 ± 1.0^a,b,c,d,e^	7.0 ± 1.4^b^	8.0 ± 0.9^c^	8.3 ± 0.9^d^	< 0.001
^*^p	> 0.05	< 0.001	> 0.05	> 0.05	> 0.05	

Note.^*^p = One-way ANOVA test with Bonferroni correction. (a) difference between Group 1 and Group 2, p < 0.001; (b) difference between Group 2 and Group 3, p < 0.01; (c) difference between Group 2 and Group 4, p < 0.001; (d) difference between Group 2 and Group 5, p < 0.001; (e) difference between basal and mid AMH and last AMH in Group 2, both p < 0.001

### Biochemical analysis

Before carboplatin and metformin were administered, basal serum AMH levels were measured by obtaining venous blood samples (1 mL) from the lateral tail veins of the rats in all groups on the first day and on the 28th day of the experiment. All AMH levels were measured. Blood samples were taken fourteen days after the last doses of carboplatin were administered, and AMH levels were measured again.

On the 28th day, anesthesia and surgical interventions were performed on all groups under sterile conditions by the same team. All rats were weighed and anesthetized via intramuscular injections of 50 mg/kg of 10% ketamine hydrochloride (Ketalar, Phizer Pharma AG) and 5 mg/kg intramuscular 2% xylazine hydrochloride (Rompun; Bayer Health Care LCC) under sterile conditions. After sterilization in the supine position, a laparotomy was performed on all rats using a 3-cm midline abdominal incision. The ovaries were excised for evaluation through histopathological and hormonal methods. After surgery, each rat was euthanized through cervical dislocation.

### Histopathologic evaluation

Histopathologic analyses of rat ovarian tissue were performed in the pathology department of the institution. For histological evaluation, ovarian tissues were processed and fixed. Serial sections of 4 μm were taken from paraffin-embedded ovary blocks using a microtome, stained with hematoxylin-eosin, and then embedded in paraffin. For statistical analysis, the follicle stage was classified according to accepted standards set by Myers et al. [14]. Ovarian follicles were evaluated by an experienced gynecological histopathologist, who was blinded to the assigned groups, as primordial, primary, secondary, and tertiary Graff follicles according to the epithelial cells surrounding the oocyte. The total follicular number was calculated per section.

### Laboratory evaluation

Blood samples for the measurement of AMH levels were centrifuged at 3,000 rpm for 10 min. The serum parts were separated and kept at -80 °C until the AMH analysis. When the measurement was to be made, it was Votex mixed (NÜVE NM 110, Ankara, Turkey). After dilution, the measurement procedure was applied. Serum AMH levels were measured using an enzyme-linked immunosorbent assay (ELISA) kit for AMH (Sunred, Biological Technology Co. HuTai Road, Shanghai, China catalog no. 201-11-1246), following the manufacturer’s recommendations. A double antibody sandwich was studied using ELISA technology. The results were expressed in ng/mL. Tissues were fixed with 150 mM KCl and centrifuged at 10,000 rpm for 30 min and supernatants were formed for MDA and CAT levels. Catalase activity was analyzed according to Aebi [15]. MDA activities were measured using the spectrophotometric method, as described by Wasowicz et al. [16].

### Statistical analysis

SPSS version 25 was used to analyze the data. The Shapiro–Wilk test was applied to evaluate whether the continuous quantitative variables were appropriately distributed in relation to the norm, and the homogeneity of variance was evaluated using the Levene test.

Regarding AMH levels, definitive statistics were shown as mean standard deviations, and the follicle counts and tissue oxidative stress markers including catalase and malondialdehyde were shown as the median (interquartile range). A one-way ANOVA test with Bonferroni correction was applied to assess the significance of the differences in the mean AMH levels across the groups. The Kruskal–Wallis test was applied to assess the significance of the differences in follicle counts and tissue oxidative stress markers. The Mann–Whitney U test was used to analyze comparisons between groups with significant median follicle numbers, and a p value < 0.05 was statistically significant.

## Results

The comparison of the baseline and 15th-day AMH values of the menstrual cycle showed no statistically significant differences between the groups, as shown in Table 1. There were statistically significant differences between the groups in AMH levels on the 28th day of the cycle. In the sham Group 1, the 28th-day AMH values were found to be statistically significantly higher than the 28th-day serum AMH values in Group 2, which was the carboplatin and saline group. Group 3, which was administered both carboplatin and metformin, had statistically significantly higher 28th-day serum AMH levels than Group 2, which was administered only carboplatin. The comparison of the AMH levels in Group 2, which was administered carboplatin and saline, and Group 4, which was administered only metformin, showed that the 28th-day AMH values were statistically significantly higher in Group 4. Group 5, which was the surgery group, had statistically significantly higher 28th -day AMH levels compared with those of Group 2, which was the carboplatin and saline group. The 28th-day AMH values in Group 2, which was administered carboplatin and saline, were statistically significantly lower than the basal and 15th-day AMH values in the same group.

**Fig. 1:**
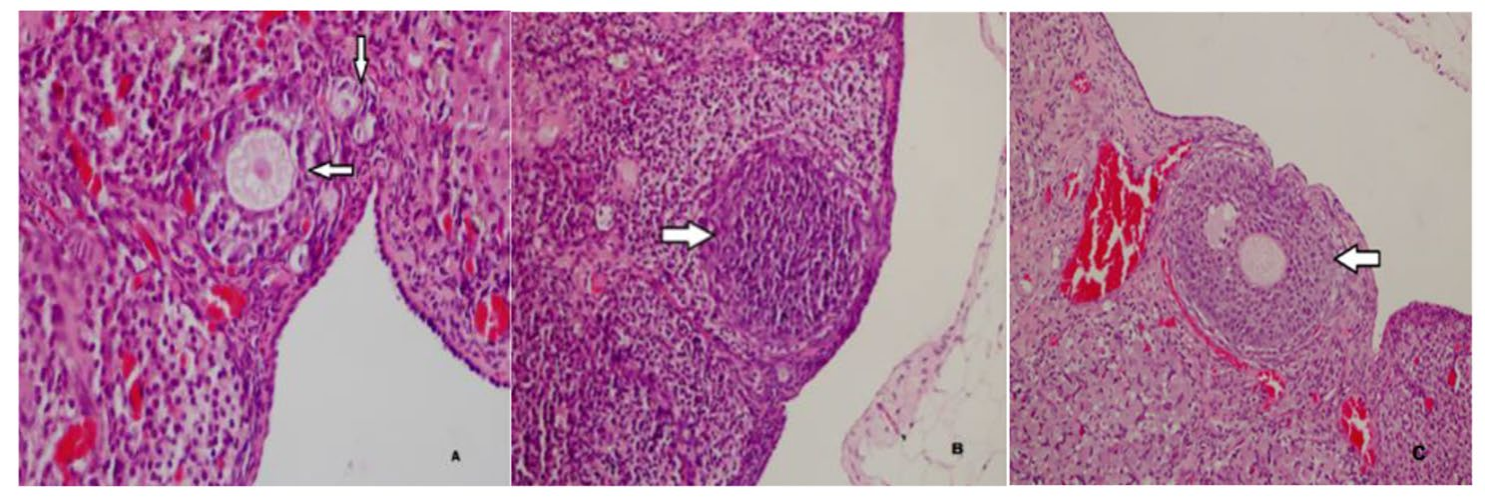
Normal primordial, primary (**A**), secondary (**B**), and tertiary (**C**) follicles in the sham group (H&E, x40, x100, x200)

**Fig. 2:**
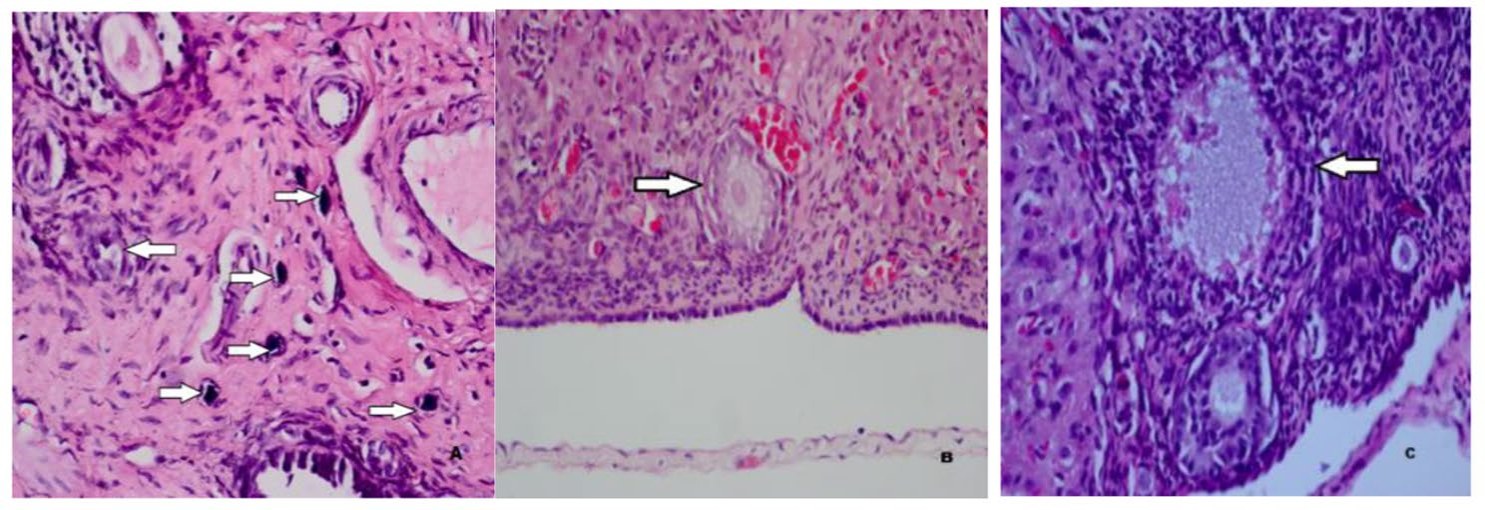
Calcified and degenerated primordial (**A**), primary (**B**) and secondary (**C**) follicles in the carboplatin-treated group (H&E, x40, x100, x200)

**Fig. 3:**
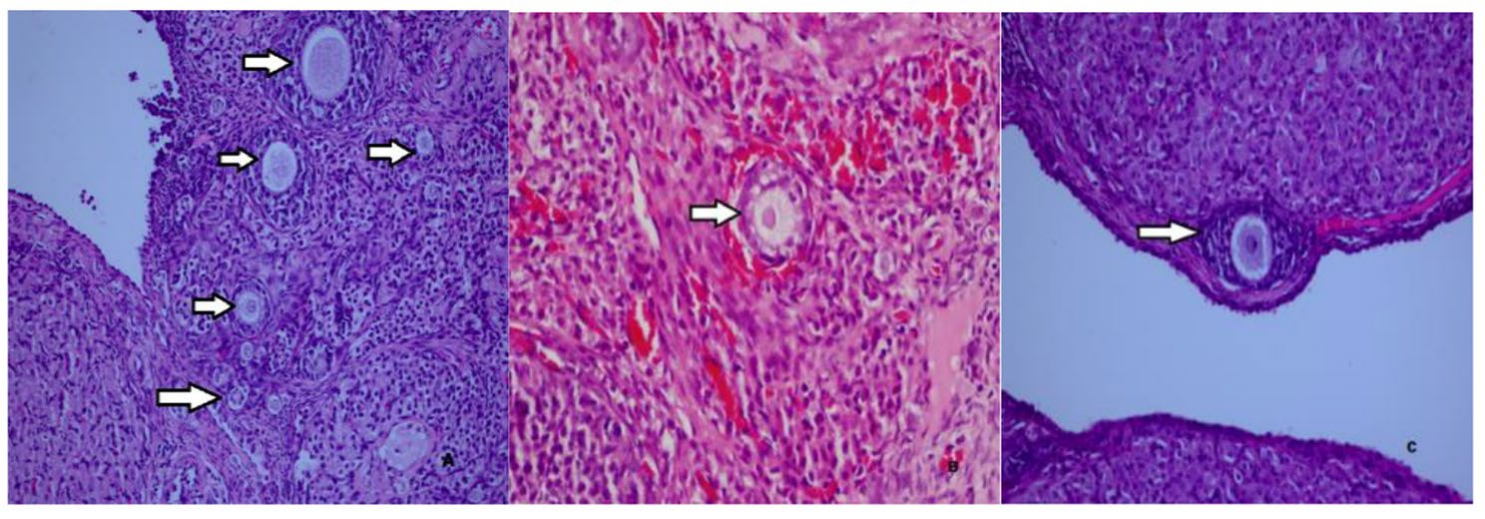
Normal primordial, primary, and secondary follicles in the ovary of a rat in in the the carboplatin plus metformin group (**A**). A normal primary follicle in the ovary of a rat in the the only metformin group (**B**). A normal secondary follicle in the ovary of a rat in the the only salin group (**C**) (H&E, x40, x100, x200)

As shown in Table 2, the number of primordial follicles in Group 2, which was administered carboplatin and saline, was statistically significantly lower than in Group 1, which was the sham group. The number of primordial follicles in Group 3, which was administered both metformin and carboplatin, was statistically significantly higher than the number of primordial follicles in Group 2, which was administered carboplatin and saline. The results showed that the number of primordial follicles was statistically significantly higher in Group 4, which was administered only metformin, than in Group 2, which was administered carboplatin and saline. The number of primordial follicles was statistically significantly higher in saline-only in Group 5 than in carboplatin-and saline in Group 2. The results also showed that the number of primary follicles was statistically significantly higher in Group 3, which was administered both metformin and carboplatin, than in Group 2, which was administered carboplatin and saline. No statistically significant difference was found between Group 2 and Group 3 in the number of tertiary follicles. The number of Graafian follicles was found to be statistically significantly higher in Group 2, which was administered carboplatin and saline, compared with Group 1, which was the sham group. The histopathological images of the follicles in each group were shown in Figs. 1 and 2, and 3.

**Table 2:** Distribution of follicle types between the groups

	Group 1 (Sham)	Group 2 (CP + Saline)	Group 3 (CP + Mtf)	Group 4 (Mtf)	Group 5 (Saline)	P
Primordial	10.4 ± 2.1^a^	5.3 ± 1.4^a,b,c,d^	9.6 ± 1.7^b^	10.0 ± 2.6^c^	10.3 ± 1.2^d^	< 0.001
Primary	4.3 ± 2.2	3.3 ± 1.4^e^	6.0 ± 1.9^e^	4.6 ± 1.7	4.4 ± 2.1	< 0.001
Secondary	3.7 ± 1.1	4.0 ± 1.5	4.1 ± 1.9	4.4 ± 1.5	4.3 ± 1.4	> 0.05
Tertiary	1.1 ± 0.9	1.4 ± 0.5^f^	1.8 ± 0.9^f^	1.3 ± 0.8	1.0 ± 0.8	> 0.05
Graafian	0.4 ± 0.5^ g^	1.0 ± 0.6^ g^	0.7 ± 0.5	1.0 ± 0.6	0.7 ± 0.8	< 0.001

Note.^*^p = Kruskal–Wallis test. (a) difference in primordial follicles between Group 1 and Group 2, p < 0.001; (b) difference in primordial follicles between Group 2 and Group 3, p < 0.001; (c) difference in primordial follicles between Group 2 and Group 4, p < 0.001; (d) difference in primordial follicles between Group 2 and Group 5, p < 0.001; (e) difference in primordial follicles between Group 2 and Group 3, p < 0.001; (f) difference in tertiary follicles between Group 2 and Group 3, p < 0.05; (g) difference in Graafian follicles between Group 1 and Group 2, p < 0.001

As shown in Table 3, the tissue catalase levels in Group 2, which was administered carboplatin and saline, were statistically significantly lower than those in Group 1, the sham group. The results showed that the tissue catalase enzyme levels in Group 3, which was administered both metformin and carboplatin, were statistically significantly higher than in Group 2, which was administered carboplatin and saline. The tissue catalase enzyme levels in Group 4, which was administered only metformin, were statistically significantly higher than in Group 2, which was administered carboplatin and saline. The results showed that the catalase enzyme levels in Group 2, which was administered carboplatin and saline, were statistically significantly lower than the tissue catalase enzyme levels in Group 5, which received only saline. The tissue malondialdehyde levels in Group 2, which was administered only carboplatin, were statistically significantly higher than those in Group 1, the sham group. The tissue malondialdehyde levels in Group 2, which was administered carboplatin and saline, were statistically significantly higher than in Group 3, which was administered both metformin and carboplatin. Similarly, the results showed that the malondialdehyde levels in Group 2, which was administered carboplatin and saline, were statistically significantly higher than in both Group 4 (metformin) and Group 5 (saline).

**Table 3:** Distributions of tissue catalase and malondialdehyde levels between groups

	Group 1 (Sham)	Grup2 (CP + Saline)	Group 3 (CP + Mtf)	Group 4 (Mtf)	Group 5 (Saline)	^*^p
Catalase	32.7 ± 2.7^a^	23.3 ± 5.1^a,b,c,d^	27.9 ± 2.2^b^	33.9 ± 4.0^c^	34.7 ± 5.4^d^	< 0.001
Malondialdehyde	87.8 ± 17.1^e^	146.7 ± 23.8^e,f,g,h^	93.9 ± 21.9^f^	96.7 ± 26.5^ g^	86.9 ± 13.2^ h^	< 0.001

Note.^*^p = Kruskal–Wallis test. (a) difference in catalase between Group 1 and Group 2, p < 0.001; (b) difference in catalase between Group 2 and Group 3, p < 0.05; (c) difference in catalase between Group 2 and Group 4, p < 0.001; (d) difference in catalase between Group 2 and Group 5, p < 0.001; (e) difference in malondialdehyde between Group 1 and Group 2, p < 0.001; (f) difference in malondialdehyde between Group 2 and Group 3, p < 0.001; (g) difference in malondialdehyde between Group 2 and Group 4, p < 0.001; (h) difference in malondialdehyde between Group 2 and Group 5, p < 0.001

## Discussion

This study aimed to determine whether metformin reduces chemotherapy-induced ovarian damage. Our results indicate that carboplatin treatment causes apparent ovarian damage which was shown by reduced existing primordial follicle pool, reduced growing follicle number and reduced AMH level. We think that metformin reduces the negative effects of carboplatin on ovarian reserve and performs this effect by affecting oxidative stress markers such as catalase and malondialdehyde at ovarian tissue [17].

Previous studies have shown that chemotherapeutic agents cause the depletion of primordial follicles, which are primarily responsible for ovarian reserve by using two main mechanisms [18]. According to activation or burn-out theory, chemotherapeutic agents stimulate dormant primordial follicles by activating the PI3K/AKT/mTOR pathway, enabling them to transform into primary oocytes and enter the growing follicle pool, resulting in the depletion of ovarian reserve [12, 19]. Another mechanism is the direction of primordial follicles to apoptosis via oxidative stress [20]. Zhou et al. [21] showed that carboplatin exerts an antineoplastic effect in ovarian cancer by inhibiting the mTOR signaling pathway. Therefore, based on this finding, it is possible that the inhibition of the mTOR signaling pathway by carboplatin may prevent the activation of primordial follicles, thus contributing positively to ovarian reserve. Because the results of the present study showed that the ovarian reserve was negatively affected, a mechanism or pathway other than the mTor pathway might have affected the ovarian reserve. According to the theory of apoptosis, the second mechanism is caused by oxidative stress, in which carboplatin reduces the ovarian reserve by directing dormant primordial follicles to apoptosis [20]. Therefore, we attributed the decrease in primordial follicles in the carboplatin-only group to the effect of carboplatin on promoting oxidative stress. The malondialdehyde levels, which are indicators of oxidative stress, were significantly higher in the group that was administered only carboplatin compared with the other groups.

Carlsson et al. [22] and Schmidt et al. [23] showed that AMH suppressed the development of primordial follicles. However, Kano et al. [24] showed that some chemotherapeutics, such as carboplatin, destroyed growing follicles and reduced the production of AMH secreted from them, confirming the results of previous studies [25]. Based on this result, in the same study, they speculated that the impediment caused by AMH to the primordial follicles would have been removed and that the primordial follicles would have been wasted by entering the growing follicle pool, resulting in a decrease in the ovarian reserve [24]. In our study, the AMH values measured on the 28th day of the menstrual cycle were lower in the carboplatin-only group compared to the basal and 15th-day AMH values in the carboplatin only group. If the decrease in AMH due to carboplatin usage stimulated the follicle pool, as shown by Kano et al. [24], we expected an increase in the number of primary and secondary follicles in the current study. However, there was no statistically significant increase in the number of primary and secondary follicles in the carboplatin-only group compared with the other groups. Therefore, this result supports our theory of oxidative damage-induced apoptosis. A previous study reported various concentrations of AMH receptors on the primordial follicle, and AMH acted in conjunction with these receptors [26]. Therefore, in the current study, the decrease in both AMH and primordial follicles in the carboplatin-only group indicates that AMH located on the primordial follicles might also be removed from the environment with follicle loss. However, this speculation needs to be proven in further research.

Several studies in the literature have shown that the antioxidant effect of metformin is effective not only in diabetic patients but also in nondiabetic pathophysiological conditions. However, the antioxidant mechanism has not been clearly explained [27]. In the current study, when we combined metformin and carboplatin, the levels of catalase, an antioxidant enzyme, increased significantly in the carboplatin plus metformin group compared with the carboplatin-saline group. At the same time, malondialdehyde, an oxidative parameter, was similarly decreased in the carboplatin plus metformin group compared with the carboplatin-saline group. The results showed that the number of primordial and primary follicles was higher in the metformin plus carboplatin group than in the carboplatin-saline group. Our results are in line with Qin X et al. [28], who observed that there were more primordial and primary follicles in mice administered metformin compared with mice that were not administered metformin. In contrast to our study, Qin X et al. [28] showed that the well-known oxidative parameters 8-OhdG and 4-HNE were significantly lower in the metformin group, and metformin rejuvenated mice ovaries by reducing the parameters of oxidative stress, which was mediated by a decrease in p16, a protein associated with aging. Sayan et al. [29] induced oxidative stress by creating a torsion–detorsion model in a rat. The results showed that malondialdehyde levels, which increased at the tissue level, were significantly reduced by a seven-day treatment with metformin. In the same study, caspase-3 levels were decreased at the tissue level with metformin treatment, which was a direct indicator of a decrease in apoptosis [29]. In our study, while carboplatin increased the oxidative stress marker malondialdehyde, it also decreased catalase, an antioxidant enzyme, and these effects were reversed with metformin treatment at the end of the 28th day.

In the current study, in the group in which metformin and carboplatin were combined, AMH levels were more highly correlated with primordial and primary follicle counts. Previous studies in the literature showed that, in most cases, the administration of metformin decreased or did not affect AMH levels, rather than increasing them [30–32]. Although the decrease in AMH levels after metformin treatment was inconsistent with our study, in other studies, decreased AMH levels were associated with PCOS. Previous research on animals showed that insulin stimulated the activation of primordial follicles, and metformin administered in PCOS cases reduced AMH levels by affecting insulin levels [27]. Based on the ability of AMH to protect primordial follicles by preventing their activation, Kano et al. [24] showed that, in mice, supraphysiological AMH given as a recombinant protein via osmotic pump or gene therapy prevented primordial follicle loss, especially due to carboplatin. Similarly, Sonigo et al. [33] showed that in mice, the gonadotoxic effect of cyclophosphamide was prevented by inhibiting the recruitment of primordial follicles when AMH treatment was added concomitantly to cyclophosphamide treatment. Although these two studies were promising in terms of oncofertility, both recommended further large-scale studies on the clinical use of AMH in humans. In our study, the rats were not administered AMH, but in the metformin–carboplatin group, the AMH levels were higher. This result partially supports Kano et al. [24] and Sonigo et al. [33]

The short-term administration of metformin, the single-dose use of the chemotherapeutic agent used in the study groups, and the lack of long-term follow-ups of AMH levels are the main limitations of this study. Due to budget constraints, another limitation of the current study was the lack of immunohistochemical staining, such as apoptosis markers. In addition, the adverse effects of metformin treatment on the course of primary disease were not analyzed.

## Conclusion

The increasing incidence of cancer in young age groups is accompanied by the long-term use of high doses of chemotherapeutics, which causes resistance to chemotherapeutics and an increased incidence of side effects [34]. Unfortunately, these side effects harm fertility. However, alternative treatment methods have been investigated to overcome the negative side effects of chemotherapeutics, such as damage to fertility [35]. The recommended treatments for fertility preservation, such as mTOR kinase inhibitors, are expensive. GnRH agonist usage is restricted to breast cancer patients in relation to the side effects of transient menopause. Moreover, AMH, as a new treatment option, is still in the experimental phase, and ovarian cryopreservation or oocyte freezing methods are invasive and expensive [36]. Therefore, these difficulties have led researchers to search for alternative combined treatment methods. According to the results of our study, as a combined agent, the addition of metformin to carboplatin treatment reduced oxidative damage and preserved primordial follicle counts. Furthermore, a high primordial follicle count was accompanied by high AMH levels. Based on these results, we recommend metformin as a fertility-preserving agent combined with carboplatin treatment. We emphasize the need for prospective randomized controlled studies in a larger series, especially in human sample populations.

## Data Availability

The datasets used and/or analysed during the current study are available from the corresponding author on reasonable request.
